# Chronic thromboembolic pulmonary hypertension

**DOI:** 10.1007/s12471-015-0667-8

**Published:** 2015-02-18

**Authors:** Michele Correale, Donato Lacedonia, Giovanna D’Andrea, Maurizio Margaglione, Matteo Di Biase, Natale Daniele Brunetti

**Affiliations:** 1 Department of Cardiology, University of Foggia, Viale L Pinto 1, 71100 Foggia, Italy; 2 Institute of Respiratory Disease, Department of Surgical and Medical Sciences, University of Foggia, Foggia, Italy; 3 Department of Clinical and Experimental Medicine, University of Foggia, Foggia, Italy

We read with great interest the review by Scholzel et al. [1], about chronic thromboembolic pulmonary hypertension (CTEPH), and especially, the sections on pathophysiology, risk factors and diagnostic work-up. However, we would like a further comment about congenital abnormalities causing hypercoagulability in these subjects. In fact, in our experience [2, 3], we have observed CTEPH patients with methylenetetrahydrofolate reductase (MTHFR) C677T polymorphism or combined deficiency of proteins C and S. Can we consider these abnormalities as predisposing factors for CTEPH? Should thrombophilia screening be performed in subjects with suspect PAH?

Could these abnormalities play a role in recurrent pulmonary hypertension after pulmonary endarterectomy?
